# Thiol–Disulfide Homeostasis and Erythrocyte Glutathione Balance in ST-Elevation Myocardial Infarction: A Prospective Case–Control Study

**DOI:** 10.3390/jcdd13090466

**Published:** 2026-09-16

**Authors:** Faruk Danış, Murat Alışık

**Affiliations:** 1 Department of Emergency Medicine, Faculty of Medicine, Bolu Abant İzzet Baysal University, Bolu 14030, Türkiye; 2 Department of Medical Biochemistry, Faculty of Medicine, Bolu Abant İzzet Baysal University, Bolu 14030, Türkiye; muratalisik@ibu.edu.tr

**Keywords:** glutathione, myocardial infarction, oxidative stress, sulfhydryl compounds, thiol–disulfide homeostasis, ST-elevation myocardial infarction

## Abstract

**Background**: ST-elevation myocardial infarction (STEMI) provokes intense oxidative stress. Serum thiol–disulfide and intracellular glutathione balances reflect oxidative status in different compartments and are rarely measured together. We compared both axes between patients with STEMI and chest-pain controls without chronic disease and examined their relation to infarct severity. **Methods**: In this prospective single-center case–control study, both axes were measured with automated assays in 90 patients with STEMI and 79 controls. Blood was drawn at first contact, before any drug and before reperfusion. Groups were compared with Welch t or Mann–Whitney U tests, with effect sizes, 95% CIs, and Benjamini–Hochberg correction. **Results**: All 10 markers differed between groups (adjusted *p* < 0.001). Patients had lower native thiol (315.0 (69.6) vs. 407.4 (63.3) µmol/L; Hedges’ *g* = −1.38) and higher disulfide (22.4 (6.8) vs. 16.9 (6.4) µmol/L; *g* = 0.82). Reduced glutathione was lower (733.2 (87.9) vs. 869.8 (83.7) µmol/L; *g* = −1.58) and oxidized glutathione higher, raising the GSSG/GSH ratio (median 0.074 vs. 0.030; *r* = 0.72). Differences persisted after adjustment for age and sex and within each sex. Within STEMI, marker–severity correlations were weak, and none survived correction. **Conclusions**: Both redox systems shift toward oxidation in STEMI. These markers were not graded indices of infarct severity, and no diagnostic or prognostic use is claimed.

## 1. Introduction

ST-elevation myocardial infarction (STEMI) results from acute, usually complete, occlusion of a coronary artery and remains a leading cause of death and disability worldwide [1,2]. Its pathophysiology is driven not only by ischemic interruption of blood supply but also by the burst of oxidative stress that accompanies ischemia and the restoration of flow. When perfusion is re-established, a surge of reactive oxygen species overwhelms antioxidant defenses and contributes to reperfusion injury, cardiomyocyte death, and adverse remodeling [3,4]. Quantifying this oxidative shift in the circulation is of continuing interest, both to understanding infarction biology and to identifying markers complementing established measures of injury and risk.

Thiol groups are principal antioxidant buffers. Under oxidative stress, reduced (native) thiols are converted to disulfide bonds, and the resulting thiol–disulfide balance can be measured in serum as a dynamic index of oxidative status. Accumulation of disulfide bonds is itself now treated as a distinct form of oxidative injury in cardiovascular disease, termed “disulfide stress” [5]. An automated assay for native thiol, total thiol, and disulfide has made this balance accessible to routine study [6], and studies have since reported altered thiol–disulfide homeostasis in many cardiovascular and inflammatory conditions, including acute coronary syndromes [7,8,9]. Glutathione is the dominant intracellular thiol antioxidant; the ratio of its reduced (GSH) to oxidized (GSSG) forms gauges intracellular redox state, and a colorimetric method quantifies these and total glutathione in erythrocytes [10].

These systems reflect different compartments. Serum thiols represent the extracellular, largely protein-bound pool, whereas erythrocyte glutathione reflects intracellular redox status. Most previous work in acute coronary syndrome examined the serum axis alone [7,8,9,11,12]. The intracellular response has received far less attention. A few coronary studies did measure a serum thiol pool together with an erythrocyte glutathione measure, using other laboratory approaches. One combined plasma protein thiols with erythrocyte reduced glutathione and glutathione peroxidase in stable coronary artery disease [13]. Another combined total thiol groups with glutathione reductase activity after primary percutaneous coronary intervention for STEMI [14]. Both reported single measures or enzyme activities rather than the partition of each axis. The reduced and oxidized fractions of the two systems therefore remain unquantified in the same coronary patients. Studies that have partitioned both axes examined chronic inflammatory and neurodegenerative disease [15,16]. A PubMed search pairing erythrocyte glutathione with thiol–disulfide homeostasis, run on 28 August 2026, returned no such study in acute coronary syndrome. Measuring both axes in the same patients therefore addresses three points that single-axis work cannot settle. The first is whether the intracellular pool moves with the serum pool during acute infarction. The second is whether the two carry the same information or separate patients from controls better in combination. The third is whether either axis tracks infarct severity. We expected both axes to shift toward oxidation, expected serum and erythrocyte measures to correlate only moderately because they sample different compartments, and expected the oxidized fractions to rise with infarct severity.

We therefore conducted a prospective case–control study with two aims: to compare serum thiol–disulfide and erythrocyte glutathione parameters between patients with STEMI and chest-pain controls; and, within the STEMI group, to examine how these redox markers relate to indices of infarct severity and early prognosis, namely peak troponin, left ventricular ejection fraction, and the GRACE and TIMI risk scores.

## 2. Materials and Methods

### 2.1. Study Design and Setting

We conducted a prospective observational case–control study in the emergency department of a tertiary-care hospital. The study followed the Declaration of Helsinki and was approved by the Non-Interventional Clinical Research Ethics Committee of Bolu Abant İzzet Baysal University Faculty of Medicine (approval no. 2024/383, dated 7 January 2025). Every participant gave written informed consent before enrollment. Reporting follows the STROBE recommendations (STROBE checklist in Supplementary Materials).

### 2.2. Participants

Cases were adults presenting to the emergency department with ST-elevation myocardial infarction, diagnosed according to the Fourth Universal Definition of Myocardial Infarction [1]. That definition requires a rise or fall of cardiac troponin with at least one value above the 99th percentile upper reference limit, together with symptoms of acute myocardial ischemia. It also requires new ST-segment elevation at the J point in two contiguous leads. The threshold is 1 mm, except in leads V2 and V3, where it is 2 mm in men aged 40 years or older, 2.5 mm in men younger than 40 years, and 1.5 mm in women. Controls were adults presenting with non-specific chest pain who had no known chronic disease (including hypertension, diabetes mellitus, hyperlipidemia, and cardiac disease) and an unremarkable physical examination. In every control the electrocardiogram was normal, and the cardiac troponin concentration lay within the reference range, so an acute coronary syndrome was ruled out before enrollment. We call this group “chest-pain controls” rather than “healthy volunteers” because they were symptomatic attenders in whom a cardiac cause had been excluded, not volunteers recruited from the community. Requiring controls to be free of chronic disease was deliberate. It keeps the measured difference attributable to the acute event rather than to a coexisting chronic oxidative burden, and it follows the design of the earlier single-axis case–control studies [7,8]. This choice has a price for interpretation, stated again in Strengths and Limitations. Because the controls carry no chronic disease, the comparison shows how STEMI differs from a disease-free state; it cannot show how STEMI differs from stable coronary artery disease or from other acute coronary syndromes. To keep redox measurements free of competing sources of oxidative stress, individuals with renal or hepatic dysfunction, active malignancy, active infection, or ongoing corticosteroid therapy were ineligible for either group. Among enrolled participants, those whose blood sample was hemolyzed or unsuitable for assay or in whom a core marker could not be measured were excluded after enrollment.

### 2.3. Variables and Measurements

For each participant we recorded age, sex, smoking status, and history of hypertension, diabetes mellitus, hyperlipidemia, or known cardiac disease. Among cases we additionally recorded the TIMI risk score, the GRACE score, and peak troponin I during the index admission (reported in ng/L). Troponin I was measured in the hospital central laboratory with an Abbott assay on an ARCHITECT i-series immunoassay analyzer (Abbott Diagnostics, Abbott Park, IL, USA). Left ventricular ejection fraction (LVEF) was taken from transthoracic echocardiography performed as part of routine clinical care, and in-hospital death was recorded. These clinical variables were not measured for the study but abstracted from hospital records. The four clinical severity measures were chosen because each is available at the bedside and indexes a different dimension of the event. Peak troponin I approximates the extent of myocardial necrosis and LVEF its functional consequence, while the TIMI and GRACE scores summarize clinical risk at presentation [17,18]. None is a direct measure of infarct size. Peak troponin depends on sampling frequency and on the timing of reperfusion, early LVEF is affected by myocardial stunning, and both risk scores are weighted by age and hemodynamic variables rather than by ischemic burden alone. These limits bear on the null correlations reported in Section 3.3.

Venous blood was drawn at first contact in the emergency department, together with the routine admission panel, before any drug was administered and before reperfusion therapy. No study sample was taken after primary percutaneous coronary intervention or thrombolysis, so the measurements describe the untreated early phase of infarction. The interval between symptom onset and sampling was not recorded. Redox status was characterized on two axes. All chemicals used in the analyses were of reagent grade and obtained from Merck (Darmstadt, Germany), and Type I ultrapure water was used throughout. Spectrophotometric analyses were performed on an ARCHITECT c8000 automated chemistry analyzer (Abbott Diagnostics, Abbott Park, IL, USA). On the serum thiol–disulfide axis, native thiol and total thiol were measured with the automated spectrophotometric method of Erel and Neşelioğlu [6]. In that method, dynamic disulfide bonds are reduced to free thiol groups with sodium borohydride to obtain total thiol. Disulfide was calculated as half the difference between total and native thiol. The disulfide/native thiol, disulfide/total thiol, and native/total thiol percentage ratios were derived from these values. For erythrocyte glutathione measurements, EDTA-anticoagulated whole blood was centrifuged, and the upper liquid phase was discarded. An equal volume of 0.9% sodium chloride was then added to replace the discarded volume. After resuspension and centrifugation, the resulting upper liquid phase was again discarded and replaced with an equal volume of 0.9% sodium chloride. This washing procedure was repeated three times, with the erythrocyte fraction retained in the tube throughout. After the final wash, the discarded saline volume was replaced with an equal volume of Type I ultrapure water to lyse the washed erythrocytes. Intracellular proteins were subsequently precipitated by adding 20% (*w*/*v*) trichloroacetic acid at a ratio of three parts erythrocyte lysate to one part acid. Following centrifugation, the resulting supernatant was collected and stored at −80 °C until analysis. Reduced glutathione (GSH) was measured directly, whereas total glutathione was determined after reduction of oxidized glutathione (GSSG) using the colorimetric method of Alışık and colleagues [10]. GSSG was calculated as half the difference between total glutathione and GSH, and the GSSG/GSH ratio was derived from these values. Measured concentrations were corrected for the dilution factors introduced during sample preparation and analysis. No additional normalization to hemoglobin concentration or erythrocyte count was applied; erythrocyte glutathione concentrations are therefore reported in µmol/L of the erythrocyte hemolysate prepared using this standardized volume-replacement procedure. Serum thiol concentrations are reported as µmol/L.

### 2.4. Sample Size

The target sample size was fixed a priori (G*Power 3.1, Heinrich Heine University Düsseldorf, Düsseldorf, Germany); two-sided α = 0.05, power 0.80), and two calculations were run. For the case–control comparison, we used the difference in total thiol reported by Kundi and colleagues in acute myocardial infarction (376 (48) µmol/L in controls versus 269 (72) µmol/L in patients) [7]. That difference corresponds to Cohen *d* = 1.75 and requires 7 participants per group. For the prespecified secondary correlation between the disulfide/total-thiol ratio and peak troponin (*r* = 0.313), the requirement was 78 patients in the case group. Recruitment was therefore governed by the correlation rather than by the group comparison, and enrollment was set above that requirement to allow for attrition. Both requirements are met by the realized analyzable sample, which comprised 90 cases and 79 controls.

### 2.5. Statistical Analysis

All analyses were performed in R (version 4.5.3; R Foundation for Statistical Computing, Vienna, Austria) within RStudio (version 2026.06.0+242; Posit Software, PBC, Boston, MA, USA), with a two-sided α of 0.05. The analysis plan, including the two-tier distinction between prespecified confirmatory and exploratory analyses, was defined a priori, before data collection and analysis. Each marker’s distribution was assessed per group with the Shapiro–Wilk test and quantile–quantile plots. Between-group comparisons used the Welch independent-samples *t*-test for approximately normally distributed markers, summarized as mean (SD) with the mean difference, its 95% confidence interval (CI), and Hedges’ *g*. Other markers were compared with the Mann–Whitney U test and summarized as median (interquartile range, IQR), with rank-biserial correlation as the effect size and its 95% CI from bootstrap resampling (2000 replicates). All tests were two-tailed. Categorical variables were compared with the χ^2^ or Fisher exact test. Because 10 redox markers were compared, the false-discovery rate across those comparisons was controlled with the Benjamini–Hochberg procedure; effect sizes with 95% CIs, rather than *p* values alone, provide the basis for interpretation.

Within the STEMI group we examined each redox marker’s association with peak troponin, LVEF, GRACE, and TIMI using Pearson or Spearman correlation according to distribution. The disulfide/total-thiol ratio versus peak troponin was the prespecified secondary target; the full marker-by-severity grid was exploratory, with Benjamini–Hochberg adjustment applied within it. As a further exploratory step, we quantified how well the leading markers separated cases from controls using the area under the receiver-operating-characteristic curve (AUC) with DeLong 95% CIs; thresholds were not prespecified, and no cut point is proposed for clinical use.

Missing marker values were to be handled by marker-wise complete-case analysis (assumed missing at random); in the analyzed sample all 10 redox markers were complete. In-hospital deaths were too few for regression modeling and are reported descriptively. Prespecified sensitivity analyses were: re-testing each between-group difference non-parametrically regardless of normality; refitting the leading markers adjusted for age and sex; excluding extreme GSSG/GSH-ratio values before re-testing that comparison; and confirming the secondary correlation under Pearson and Spearman methods. Extreme was defined in the analysis plan as any GSSG/GSH ratio above the third quartile plus three interquartile ranges of the pooled distribution. The analysis plan did not prespecify primary markers. The initial adjusted analyses focused on the four markers showing the largest observed effect sizes; to reduce the potential for selective reporting, adjusted models were subsequently applied to all ten markers. Two additional analyses were conducted post hoc: marker–severity correlations were repeated using log-transformed peak troponin, and the areas under the receiver-operating characteristic curves of the individual markers were compared pairwise using the DeLong test. R and package versions were recorded (sessionInfo).

Three additional analyses were performed post hoc. First, the age- and sex-adjusted linear models, initially applied to four markers, were extended to all ten markers and additionally repeated with smoking included as a covariate (Table S1). Second, case–control comparisons were repeated separately in men and women, and marker concentrations were also compared between men and women within each group (Tables S2 and S3). Third, logistic models combining one serum and one erythrocyte marker were compared with the corresponding single-marker models using the DeLong test for correlated areas under the receiver operating characteristic curves; correlations between the serum and erythrocyte axes were also examined (Table S4). Additional adjustment for comorbidities was not performed because hypertension, diabetes mellitus, hyperlipidemia, and known cardiac disease were absent in all controls by eligibility criteria, resulting in quasi-complete separation and precluding reliable estimation of the corresponding logistic regression coefficients.

## 3. Results

### 3.1. Participant Flow and Baseline Characteristics

**Figure 1:**
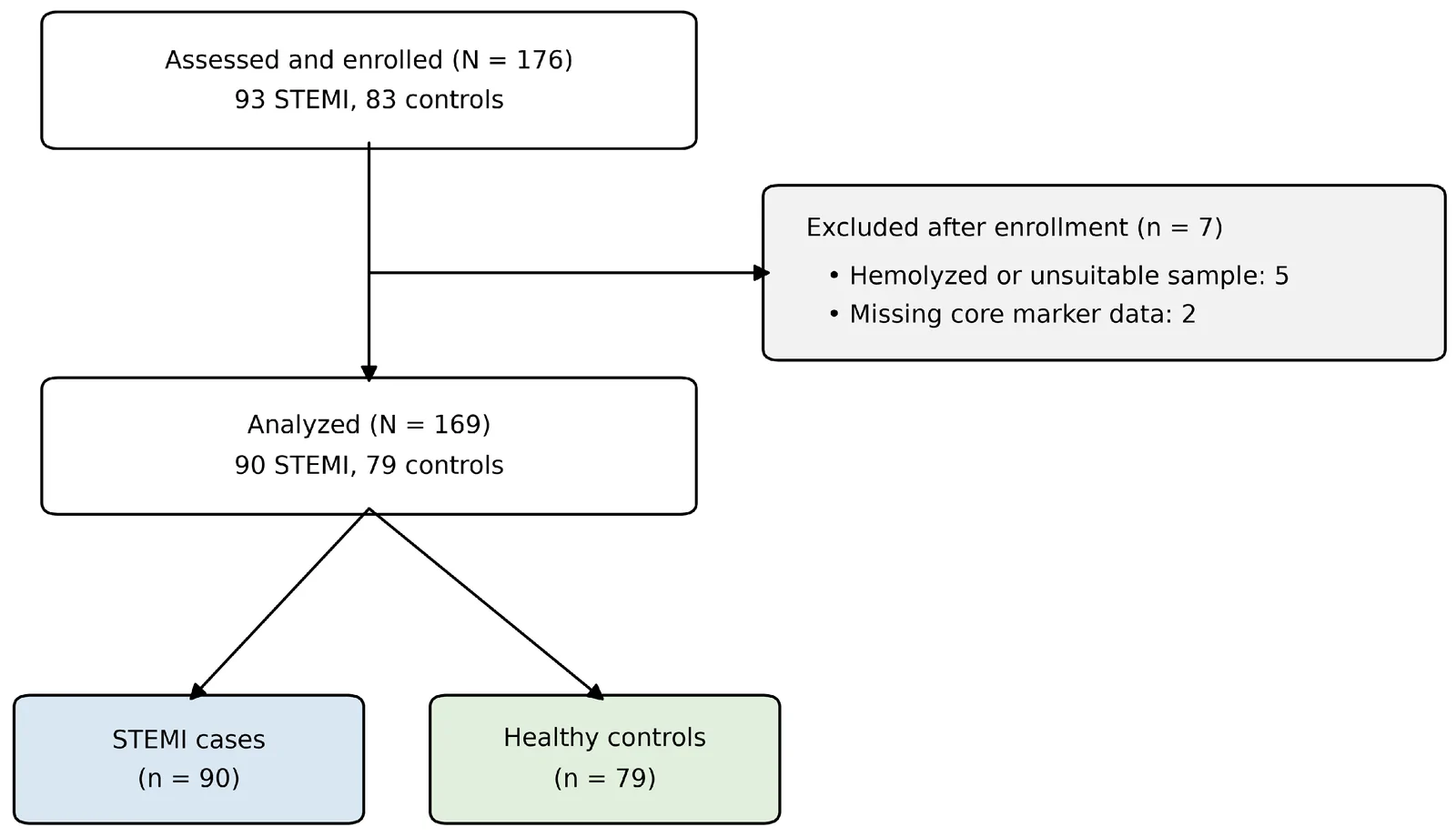
Participant flow. Of 176 enrolled, 7 were excluded after enrollment (5 with hemolyzed or unsuitable samples, 2 missing core data), leaving 169 for analysis (90 with ST-elevation myocardial infarction, 79 controls).

A total of 176 participants were enrolled (93 STEMI, 83 controls); 7 were excluded after enrollment because the blood sample was hemolyzed or unsuitable (5) or a core marker could not be measured (2), leaving 169 for analysis (90 STEMI, 79 controls) (Figure 1). The groups differed on several baseline characteristics (Table 1). Cases and controls were of similar age (54.4 (10.5) vs. 53.8 (9.8) years; *p* = 0.733), but cases were more often male (75.6% vs. 19.0%; *p* < 0.001). Controls were free of chronic disease by eligibility criteria, whereas among cases hypertension was present in 34.4%, hyperlipidemia in 28.9%, known cardiac disease in 26.7%, and diabetes mellitus in 15.6% (*p* < 0.001 versus controls for each). Smoking, which was not an exclusion criterion, did not differ between groups (33.3% vs. 25.3%; *p* = 0.332).

### 3.2. Thiol–Disulfide and Glutathione Differences Between STEMI and Controls

**Figure 2:**
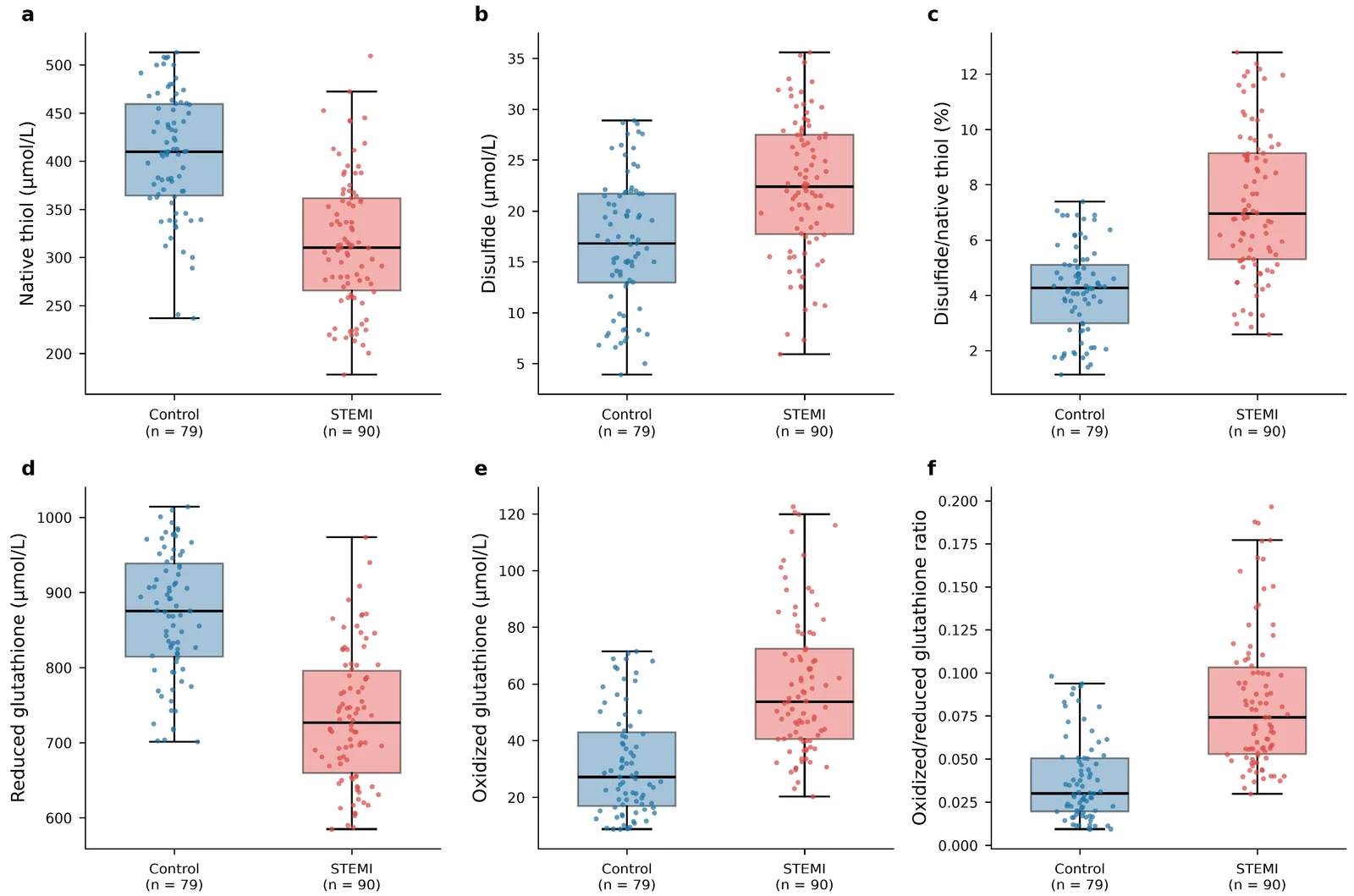
Serum thiol–disulfide and erythrocyte glutathione markers in ST-elevation myocardial infarction versus controls: native thiol (**a**), disulfide (**b**), disulfide/native-thiol ratio (**c**), reduced glutathione (**d**), oxidized glutathione (**e**), and oxidized-to-reduced glutathione ratio (**f**). Boxes show the median and interquartile range, whiskers 1.5 times that range, and each point one participant.

All 10 redox markers differed between cases and controls, and every difference remained significant after Benjamini–Hochberg correction (adjusted *p* < 0.001 each; Table 2, Figure 2). Both systems moved in the same direction: reduced species were depleted in STEMI and oxidized species accumulated.

On the serum thiol–disulfide axis, native thiol was markedly lower in cases (315.0 (69.6) vs. 407.4 (63.3) µmol/L; Hedges’ *g* = −1.38, 95% CI −1.72 to −1.04), as was total thiol (359.7 (72.8) vs. 441.2 (67.0) µmol/L; *g* = −1.16, −1.48 to −0.83). Disulfide moved the other way, rising to 22.4 (6.8) µmol/L in cases from 16.9 (6.4) µmol/L in controls (*g* = 0.82, 0.50 to 1.14). As native thiols decreased and disulfide increased, the proportion of oxidized species rose accordingly: the disulfide/total-thiol ratio reached 6.1% (IQR 4.8–7.7) in cases versus 3.9% (2.8–4.6) in controls (rank-biserial *r* = 0.69, 0.57 to 0.80), the disulfide/native-thiol ratio was 7.0% (5.3–9.1) versus 4.3% (3.0–5.1) (*r* = 0.69, 0.57 to 0.80), and the native/total-thiol ratio fell to 87.8% (84.6–90.4) from 92.1% (90.7–94.4) (*r* = −0.69, −0.80 to −0.57).

The erythrocyte glutathione axis showed the same pattern. Reduced glutathione was much lower in cases (733.2 (87.9) vs. 869.8 (83.7) µmol/L; Hedges’ *g* = −1.58, 95% CI −1.93 to −1.23, the largest single effect), total glutathione moderately lower (852.2 (85.0) vs. 933.4 (81.0) µmol/L; *g* = −0.97, −1.29 to −0.65), and oxidized glutathione was markedly higher (median 53.9 (IQR 40.5–72.4) vs. 27.2 (17.0–42.9) µmol/L; rank-biserial *r* = 0.64, 0.52 to 0.76). Expressed as the oxidized-to-reduced fraction, the GSSG/GSH ratio was correspondingly higher in cases, with a median 0.074 (0.053–0.103) versus 0.030 (0.019–0.050) (*r* = 0.72, 0.61 to 0.83). Both axes therefore moved in the direction expected when antioxidant reserve is consumed, reduced species falling and oxidized species rising in serum and inside the red cell alike.

### 3.3. Association of Redox Markers with Clinical Severity Measures

**Figure 3:**
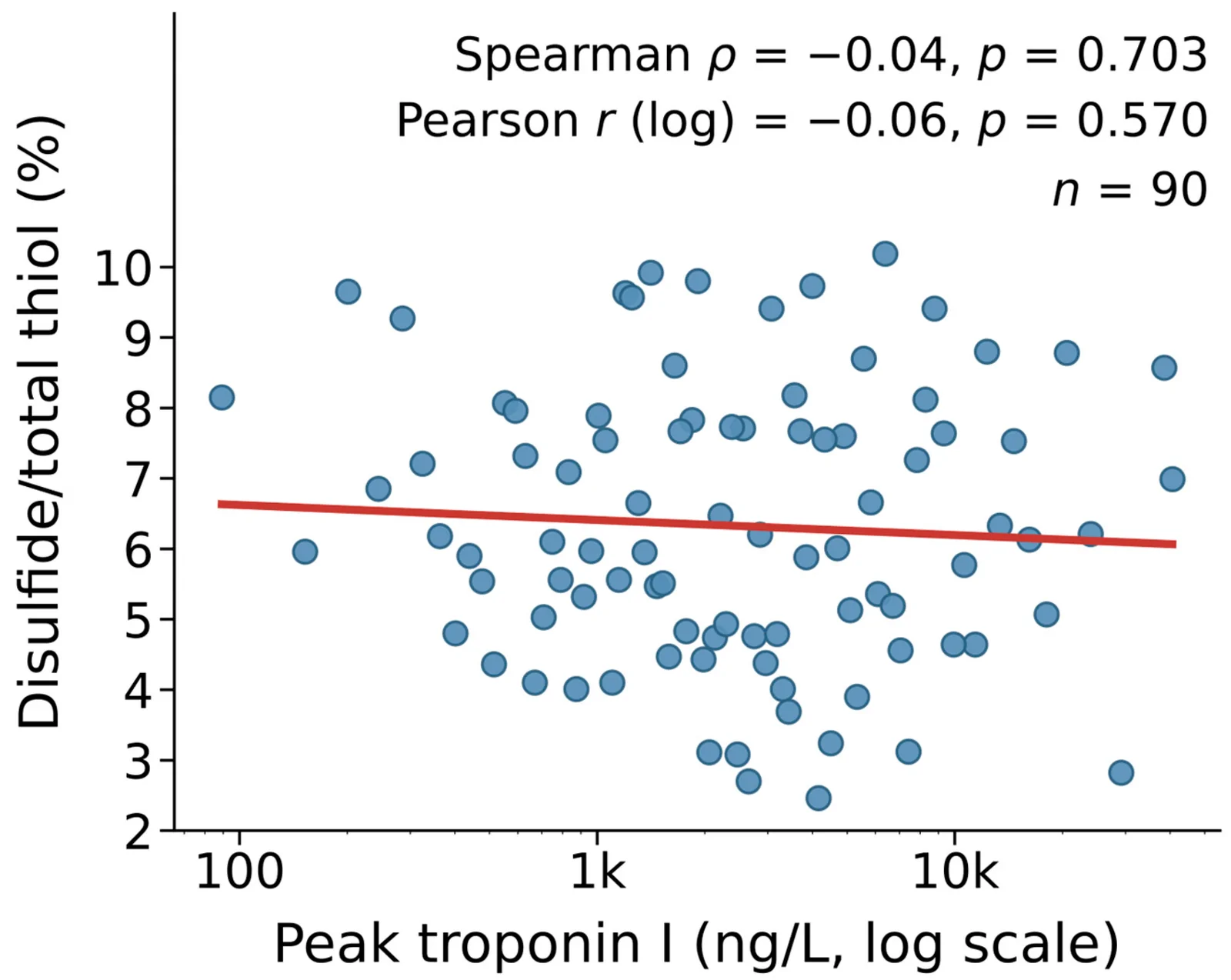
Association between the disulfide/total-thiol ratio and peak troponin I (ng/L, logarithmic scale) within the ST-elevation myocardial infarction group (prespecified secondary target, exploratory). The line is an ordinary-least-squares fit for visual guidance; the statistic is the Spearman correlation, which a logarithmic transformation of the x axis leaves unchanged. Each point represents one patient with ST-elevation myocardial infarction.

Within the STEMI group, correlations between the redox markers and the severity indices were weak, and none survived false-discovery-rate correction across the 40-pair grid; these associations are therefore reported as exploratory. The prespecified secondary target was the disulfide/total-thiol ratio against peak troponin I, and it was essentially null (Spearman *ρ* = −0.04; *p* = 0.703; *n* = 90; Figure 3) against the *r* = 0.313 anticipated from earlier work. Peak troponin I in the STEMI group ranged from 89 to 40,594 ng/L (median 2423 ng/L, IQR 1020–5758). No marker–severity correlation reached nominal significance; the closest was oxidized glutathione with LVEF (*ρ* = −0.18; *p* = 0.096), with all other raw *p* values being higher. No correlation with peak troponin exceeded *ρ* = 0.15, and none with GRACE or TIMI exceeded *ρ* = 0.13. Because peak troponin was strongly right-skewed, the correlations were repeated against log-transformed peak troponin. A monotone transformation leaves rank correlations unchanged, so the Spearman coefficients are identical. The Pearson coefficients on the log scale also remained weak. The largest was 0.13, for reduced glutathione, and none reached significance after correction (all adjusted *p* ≥ 0.66; Table S5a). Of the 90 patients with STEMI, 9 died in hospital (10.0%). Deaths were too few for prognostic modeling, so this outcome is reported descriptively. Within the range of severity sampled here, these markers behaved as signals of the infarction state rather than as graded measures of its size.

### 3.4. Discrimination Between STEMI and Controls (Exploratory)

**Figure 4:**
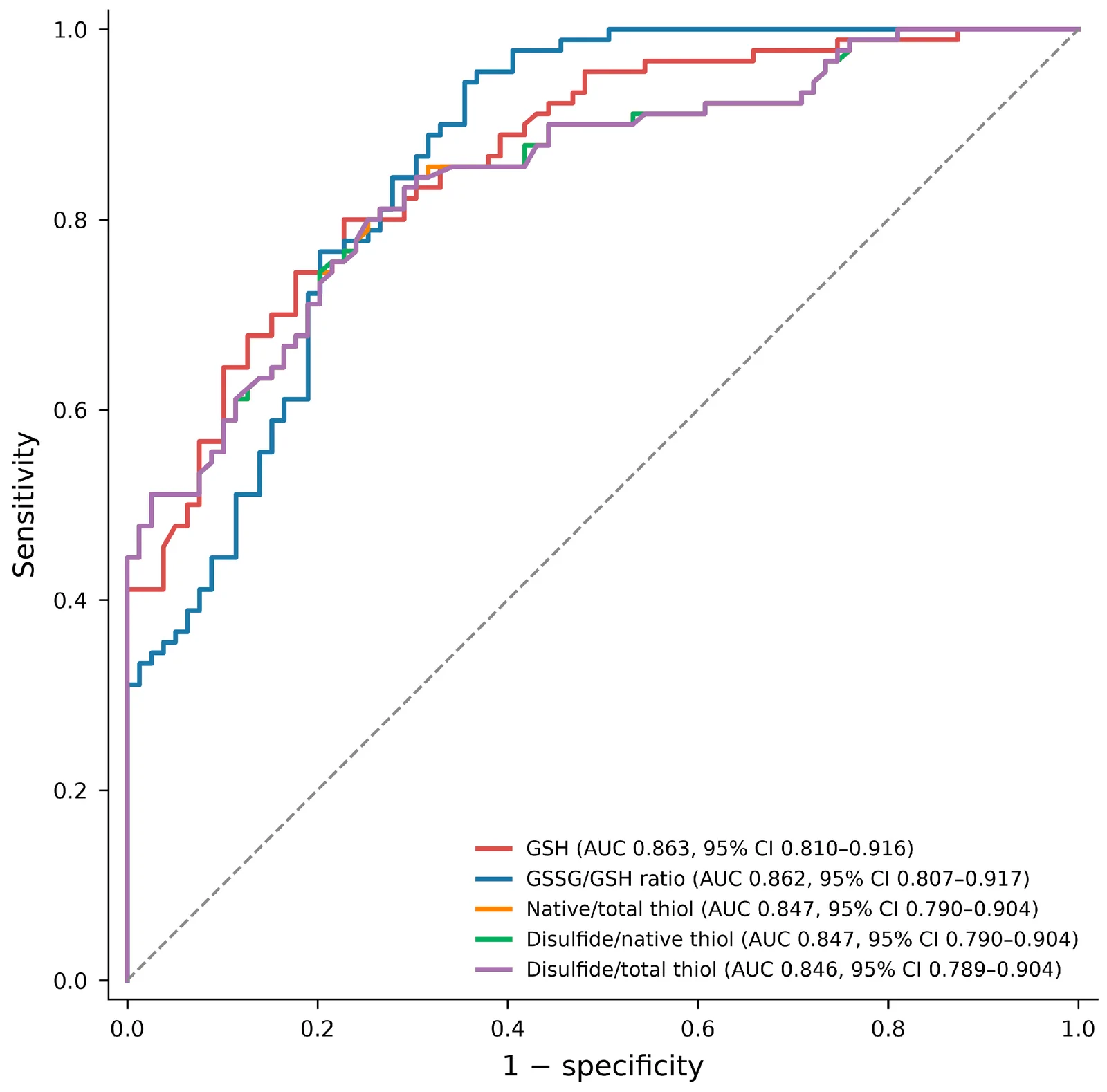
Receiver-operating-characteristic curves discriminating ST-elevation myocardial infarction from control participants for the five markers with the highest areas under the curve. The analysis is exploratory: the curves were fitted and evaluated in the same sample, and no threshold was prespecified. Pairwise comparison separated these five markers from the weakest ones but could not distinguish them from one another. The diagonal dashed line is the line of no discrimination (area under the curve 0.5).

Reflecting the large between-group differences, several markers separated cases from controls with high discrimination on receiver-operating-characteristic analysis (Figure 4). The highest area under the curve was that of reduced glutathione (0.863; 95% CI 0.810–0.916), with the GSSG/GSH ratio (0.862), the native/total-thiol and disulfide/native-thiol ratios (0.847 each), the disulfide/total-thiol ratio (0.846), and native thiol (0.835) close behind. The ranking alone does not establish that one marker discriminates better than another, so all 45 pairwise comparisons of the single-marker curves were tested with the DeLong test for correlated curves. Reduced glutathione separated cases from controls no better than did the GSSG/GSH ratio (0.863 versus 0.862; *p* = 0.97) or any of the three thiol ratio measures, native thiol or oxidized glutathione (adjusted *p* ≥ 0.30). It did exceed total glutathione (0.757; adjusted *p* < 0.001) and disulfide (0.719; adjusted *p* = 0.013). Of the 45 comparisons, 8 reached significance after correction, and none of them lay among the five leading markers, whose curves were statistically indistinguishable from one another (Table S5b). Combining the two axes increased separation. Reduced glutathione with native thiol reached an AUC of 0.916 (0.876–0.956), exceeding reduced glutathione alone (DeLong *p* = 0.004) and native thiol alone (*p* < 0.001). Pairing the two oxidized fractions, the GSSG/GSH ratio with the disulfide/total-thiol ratio, gave 0.930 (0.894–0.965; *p* = 0.014 versus reduced glutathione alone; Table S4). These combined models were fitted post hoc in the same sample, without resampling or external validation. They therefore quantify separation and complementarity between the two axes in this dataset, not the performance of a diagnostic test. No cut point is proposed.

### 3.5. Sensitivity Analyses

The main findings were robust. Re-testing the normally distributed markers (native thiol, total thiol, disulfide, reduced and total glutathione) with the Mann–Whitney U test left every between-group difference significant (all *p* < 0.001). Extending the age- and sex-adjusted linear models from the four leading markers to all ten left every case–control difference significant. Reduced glutathione stood at adjusted β = −127.6 µmol/L (95% CI −159.4 to −95.8), oxidized glutathione at 28.7 (20.5 to 36.9), native thiol at −85.0 (−109.6 to −60.5), disulfide at 6.3 (3.8 to 8.7) and the GSSG/GSH ratio at 0.05 (0.03 to 0.06). Adding smoking to those models changed no estimate materially (Table S1). The difference also held within each sex: all ten markers separated cases from controls in men (15 controls, 68 cases) and in women (64 controls, 22 cases), with every adjusted *p* being below 0.02 (Table S2). Inside each group, no marker differed between men and women after correction, with the smallest adjusted *p* value being 0.643 (Table S3). Serum and erythrocyte markers correlated moderately with one another and native thiol with reduced glutathione at Spearman *ρ* = 0.45 (*p* < 0.001), so the two axes were related without being redundant (Table S4). The prespecified rule for extreme GSSG/GSH-ratio values was any value above the third quartile plus three interquartile ranges of the pooled distribution. That threshold is 0.241. No observation exceeded it. No participant was therefore excluded, and this analysis is identical to the primary comparison (rank-biserial *r* = 0.72, 95% CI 0.61 to 0.83; *p* < 0.001). The secondary correlation was concordant under Pearson and Spearman methods.

## 4. Discussion

In this prospective case–control study, we measured oxidative balance in STEMI on two complementary axes at once: the extracellular serum thiol–disulfide couple and the intracellular erythrocyte glutathione pool. Both were shifted toward oxidation relative to the chest-pain controls. The separation was large and internally consistent. Native and total thiol was lower, while disulfide and the disulfide/thiol ratios were higher. Reduced glutathione was lower, total glutathione only modestly so, and oxidized glutathione rose. The GSSG/GSH ratio was markedly higher. Within STEMI, however, these markers tracked severity only weakly, and no marker–severity correlation survived correction.

The thiol–disulfide changes align with the literature on acute coronary syndromes, in which the reduced thiol pool contracts and the oxidized fraction expands as antioxidant reserve is consumed [11,12]. A comparable shift is documented in other acute ischemic and ischemia–reperfusion settings, including acute ischemic stroke and cardiac surgery with cardiopulmonary bypass [19,20,21]. That breadth suggests a general feature of acute ischemic injury rather than a STEMI-specific artifact. Cohorts with non-ST-elevation acute coronary syndrome show lower native and total thiol and higher disulfide/thiol ratios than controls, with native thiol falling as the GRACE score and thrombus burden rise [7,8,9]. Our data reproduce the direction of these group differences throughout, that is, the fall in native and total thiol and the rise in the disulfide fractions. The severity association reported in those cohorts was not reproduced here: none of the correlations with peak troponin, LVEF, GRACE or TIMI reached significance in our sample (Section 3.3).

One difference of degree is worth noting. Where some non-ST-elevation cohorts found absolute disulfide essentially unchanged between patients and controls, we observed higher absolute disulfide in STEMI (*g* = 0.82; 95% CI 0.50 to 1.14) alongside the expected ratio shift [8,9]. Sampling time may contribute to this, although it cannot be verified across studies. Our samples were drawn at first contact, before any drug and before reperfusion, which places them at the earliest measurable point of the event. Reports from other cohorts rarely state when blood was taken relative to treatment, so we cannot confirm that they sampled later; if they did, they would capture a later phase of a balance that moves within hours. Severity at presentation and the interval from symptom onset also differ between cohorts. A further possibility is that the more complete, usually transmural occlusion of STEMI carries a larger ischemic and thrombotic burden. We could not test it because thrombus burden and infarct territory were not recorded, so we offer it as a hypothesis rather than as an explanation supported by our data. The concordance of ratio changes across studies indicates the oxidative signal is read most reliably from the balance between reduced and oxidized species.

The glutathione findings extend the picture to the intracellular compartment: reduced and total glutathione were lower and oxidized glutathione higher in cases, markedly raising the GSSG/GSH ratio. Because glutathione is the principal thiol antioxidant of the erythrocyte, the oxidative challenge of acute infarction is evidently not confined to circulating serum proteins but registered inside circulating cells. To our knowledge combined serum thiol–disulfide and erythrocyte glutathione assessment has previously been applied to chronic inflammatory and neurodegenerative conditions, rheumatoid arthritis and Alzheimer’s disease, where both axes likewise shifted toward oxidation together [15,16]. We apply this paired approach to STEMI. That both axes moved in the same direction and reduced glutathione carried the largest effect and strongest discrimination strengthens the inference of a genuine systemic redox shift rather than of an artifact of one assay or compartment.

The size of the shift is consistent with thiol chemistry during ischemia and reperfusion. Reactive oxygen species oxidize protein cysteine residues to disulfides, so the reduced pool contracts while the oxidized pool grows, and the ratio between them moves faster than either absolute concentration [5,22]. Glutathione is the erythrocyte’s main defense. It is consumed as peroxides are reduced by glutathione peroxidase, and GSSG accumulates once the recycling capacity of glutathione reductase is exceeded [10]. Red cells passing through an ischemic and then reperfused bed meet that peroxide load directly, which may explain why the intracellular pool registers the event rather than being spared.

That the same markers separating cases from controls so sharply correlated only weakly with troponin, LVEF, GRACE, and TIMI within the STEMI group deserves emphasis. It suggests these measures behave more as qualitative signals of the infarction state than as graded indices of its magnitude, at least over the severity range and sample size available here. No individual marker–severity association reached nominal significance, and the prespecified association between the disulfide/total-thiol ratio and peak troponin was essentially null (*ρ* = −0.04), unlike the moderate correlation reported previously [7]. These correlations are hypothesis-generating, and the AUC values describe separation in this sample, not diagnostic thresholds.

Three explanations for the null associations are worth separating. The first is timing. In the largest STEMI series to address this question, systemic free thiols were measured serially. The associations with infarct size and ejection fraction were carried by the change over the first 24 h rather than by the concentration at presentation [23]. A single early sample, which is what we drew, cannot capture that dynamic. The second is what the severity indices measure. Peak troponin, early LVEF and the risk scores are indirect and partly non-ischemic in origin, and other STEMI cohorts using different redox panels have related their markers to treatment response and inflammation rather than to infarct size [24,25]. The third is power. With 90 cases the study had 86% power for the correlation of 0.313 anticipated from earlier work, so the study was unlikely to miss an effect of that size. Power was only 47% for a correlation of 0.20, however, and 29% for one of 0.15. Weak true associations therefore remain compatible with our data [7,26].

The sex imbalance between the groups was large: 75.6% of cases and 19.0% of controls were men. Two analyses argue that it did not create the redox difference. Adjustment for age and sex left all ten markers significant with estimates close to the unadjusted ones (Table S1), and the case–control gap was reproduced separately in men and in women (Table S2). Within each group, men and women did not differ on any marker after correction (Table S3). Sex behaves here as a marker of STEMI development rather than as a determinant of thiol or glutathione status, which is consistent with the small sex-related differences reported in redox studies of atherosclerotic disease [27].

Whether the two axes together add anything beyond a single measurement can be answered directly. They were related but not interchangeable. Serum and erythrocyte markers correlated at *ρ* near 0.45, so about a fifth of the variance was shared. Combining one marker from each compartment raised the area under the curve from 0.863 to 0.916, a difference the DeLong test indicated was unlikely to be noise (*p* = 0.004). We read this as biological complementarity rather than as a diagnostic model. The comparison was made in the sample that generated it, no threshold was fixed, and STEMI is diagnosed by electrocardiography and troponin, not by redox markers. The practical implication is for study design: a panel spanning both compartments describes the redox response of acute infarction more completely than does either axis alone.

Several factors other than infarction move these markers and deserve explicit mention. Smoking lowers native and total thiol and raises the disulfide fractions in otherwise healthy people [28]. Diabetes mellitus and its chronic complications shift thiol–disulfide homeostasis in the same direction [29], and statin therapy alters systemic thiol status and antioxidant enzyme activity in advanced atherosclerosis [27]. The glutathione axis responds to the same exposures when it is measured by other techniques: glutathione synthesis is reduced in type 2 diabetes, more so with microvascular complications [30], and blood glutathione and glutathione-dependent enzyme activities differ with smoking and with lipid profile in otherwise healthy adults [31]. Acute-phase processes matter as well since albumin carries most of the serum thiol pool and falls during acute illness [22]. Our design limits some of these influences and leaves others open. Smoking did not differ between groups (33.3% versus 25.3%), and adding it to the adjusted models changed nothing (Table S1). No drug had been given when the samples were drawn. In-hospital treatment therefore cannot have contributed. Chronic medication taken before admission was not recorded, however, and cases and controls differed by design in hypertension, diabetes and hyperlipidemia, so a contribution from long-standing metabolic burden cannot be separated from the acute event.

### Strengths and Limitations

Strengths include the prospective design and the paired measurement of two redox axes with validated automated assays. Sampling was fixed at first contact, before any drug and before reperfusion, which removes in-hospital treatment as an explanation for the differences observed. The analysis plan was fixed before outcome relationships were inspected. Effect sizes with confidence intervals are reported alongside false-discovery-rate control, and the sensitivity analyses, non-parametric re-testing, adjusted models for all ten markers, sex-stratified comparisons and outlier exclusion, left the conclusions unchanged. That the case–control differences were independent of the sex imbalance is reassuring, with cases being more often male.

Several limitations temper the conclusions. This was a single-center study of 169 participants. The findings need confirmation elsewhere. Because controls were required to be free of chronic disease, the two groups differed in cardiovascular risk profile by design. Adjustment for age and sex did not alter the redox differences, but comorbidity itself could not be adjusted for. No control had hypertension, diabetes mellitus, hyperlipidemia or known cardiac disease, so those models are not estimable. The comparison isolates STEMI from health rather than STEMI from coronary artery disease in general, and patients with non-ST-elevation acute coronary syndrome or stable coronary disease would be the informative next comparator [27]. Redox markers were measured at a single early time point, so their trajectory and recovery cannot be described, and the interval from symptom onset to sampling was not recorded. Chronic pre-admission medication was not collected. In-hospital deaths were infrequent (9 of 90 cases), precluding prognostic modeling and leaving the relationship with hard outcomes unresolved. The design supports association only. A case–control comparison at a single time point cannot show that infarction caused the oxidative shift, and the moderate correlation between the two axes is compatible with a shared systemic driver but does not establish that they are mechanistically coupled. The discrimination analyses were exploratory, fitted and evaluated in the same sample without prespecified thresholds or external validation, so they support no diagnostic claim. Finally, the weak marker–severity correlations may reflect limited power for associations of this size within the case group. Larger multicenter cohorts with serial sampling and clinical endpoints are needed to establish whether these measures carry prognostic value [23,32,33].

## 5. Conclusions

In patients with STEMI, both the serum thiol–disulfide couple and the erythrocyte glutathione pool were shifted toward the oxidized state relative to chest-pain controls in whom a cardiac cause had been excluded. Effect sizes were large and internally consistent across both axes: reduced species were depleted and oxidized species accumulated. The shift survived adjustment for age and sex. It was present in men and in women separately. Measuring both compartments together shows that the oxidative shift is detectable in the circulation and inside the red cell at the same time, and the two axes proved complementary rather than interchangeable. Within the STEMI group, however, these markers correlated only weakly with indices of infarct severity, and no association survived correction for multiple testing. The discriminative values observed were exploratory and describe case–control separation in this sample; they do not support diagnostic or prognostic use, and no threshold is proposed. Whether combined thiol–disulfide and glutathione assessment adds clinical information in STEMI must be settled by larger multicenter studies with serial sampling, an acute coronary syndrome comparator group and hard endpoints.

## Figures and Tables

**Table 1:** Baseline characteristics of the study population.

Characteristic	Controls (*n* = 79)	STEMI (*n* = 90)	*p* Value
Age, years ^a^	53.8 (9.8)	54.4 (10.5)	0.733
Male sex, *n* (%) ^b^	15 (19.0)	68 (75.6)	<0.001
Current smoking, *n* (%) ^b^	20 (25.3)	30 (33.3)	0.332
Hypertension, *n* (%) ^b^	0 (0.0)	31 (34.4)	<0.001
Diabetes mellitus, *n* (%) ^b^	0 (0.0)	14 (15.6)	<0.001
Hyperlipidemia, *n* (%) ^b^	0 (0.0)	26 (28.9)	<0.001
Known cardiac disease, *n* (%) ^b^	0 (0.0)	24 (26.7)	<0.001

^a^ Mean (SD); Welch *t*-test. ^b^ Number (%); χ^2^ test. Abbreviations: SD, standard deviation; STEMI, ST-elevation myocardial infarction.

**Table 2:** Serum thiol–disulfide and erythrocyte glutathione parameters in patients with ST-elevation myocardial infarction versus controls.

Marker	Controls (*n* = 79)	STEMI (*n* = 90)	Effect Size (95% CI)	*p* Value	Adjusted *p* Value
Native thiol, µmol/L ^a^	407.4 (63.3)	315.0 (69.6)	Hedges’ g −1.38 (−1.72 to −1.04)	<0.001	<0.001
Total thiol, µmol/L ^a^	441.2 (67.0)	359.7 (72.8)	Hedges’ g −1.16 (−1.48 to −0.83)	<0.001	<0.001
Disulfide, µmol/L ^a^	16.9 (6.4)	22.4 (6.8)	Hedges’ g 0.82 (0.50 to 1.14)	<0.001	<0.001
Disulfide/native thiol ratio, % ^b^	4.3 (3.0–5.1)	7.0 (5.3–9.1)	Rank-biserial r 0.69 (0.57 to 0.80)	<0.001	<0.001
Disulfide/total thiol ratio, % ^b^	3.9 (2.8–4.6)	6.1 (4.8–7.7)	Rank-biserial r 0.69 (0.57 to 0.80)	<0.001	<0.001
Native thiol/total thiol ratio, % ^b^	92.1 (90.7–94.4)	87.8 (84.6–90.4)	Rank-biserial r −0.69 (−0.80 to −0.57)	<0.001	<0.001
Reduced glutathione, µmol/L ^a^	869.8 (83.7)	733.2 (87.9)	Hedges’ g −1.58 (−1.93 to −1.23)	<0.001	<0.001
Total glutathione, µmol/L ^a^	933.4 (81.0)	852.2 (85.0)	Hedges’ g −0.97 (−1.29 to −0.65)	<0.001	<0.001
Oxidized glutathione, µmol/L ^b^	27.2 (17.0–42.9)	53.9 (40.5–72.4)	Rank-biserial r 0.64 (0.52 to 0.76)	<0.001	<0.001
Oxidized/reduced glutathione ratio ^b^	0.030 (0.019–0.050)	0.074 (0.053–0.103)	Rank-biserial r 0.72 (0.61 to 0.83)	<0.001	<0.001

^a^ Mean (SD); Welch *t*-test; effect size Hedges’ *g*. ^b^ Median (interquartile range); Mann–Whitney U test; effect size rank-biserial correlation with bootstrap 95% CI. Adjusted *p* values are Benjamini–Hochberg false-discovery-rate adjusted across the 10 marker comparisons. All tests were two-tailed. Abbreviations: see Table 1; CI, confidence interval.

## Data Availability

The data presented in this study are available on request from the corresponding author. The data are not publicly available because they consist of individual-level clinical and laboratory records collected at a single center, and their release is restricted for reasons of participant privacy.

## Supplementary Materials

The following supporting information can be downloaded at https://www.mdpi.com/article/10.3390/jcdd13090466/s1: Table S1: Between-group differences in all ten redox markers after adjustment for age and sex, and after additional adjustment for smoking (linear models, *n* = 169); Table S2: Sex-stratified comparison of all ten redox markers between patients with ST-elevation myocardial infarction and controls; Table S3: Redox markers by sex within each group; Table S4: Exploratory discrimination of single-axis and two-axis marker combinations, and correlation between the two axes; Table S5a: Correlations between the redox markers and peak troponin I on the raw and the log-transformed scale, within the ST-elevation myocardial infarction group (*n* = 90); Table S5b: Pairwise comparison of the single-marker areas under the curve against the marker with the highest value (DeLong test). STROBE checklist.

## Abbreviations

The following abbreviations are used in this manuscript:

AUC	area under the receiver-operating-characteristic curve
CI	confidence interval
GRACE	Global Registry of Acute Coronary Events
GSH	reduced glutathione
GSSG	oxidized glutathione
IQR	interquartile range
LVEF	left ventricular ejection fraction
SD	standard deviation
STEMI	ST-elevation myocardial infarction
STROBE	Strengthening the Reporting of Observational Studies in Epidemiology
TIMI	Thrombolysis in Myocardial Infarction
