# Supplementary material for: Thiol–Disulfide Homeostasis and Erythrocyte Glutathione Balance in ST-Elevation Myocardial Infarction: A Prospective Case–Control Study
Source: J Cardiovasc Dev Dis. 2026 Sep 16;13(9):466. doi: 10.3390/jcdd13090466 (PMC13607282; doi:10.3390/jcdd13090466)
Supplement: Supplementary file 1 [file jcdd-13-00466-s001.zip › STROBE checklist.pdf]

STROBE Statement — checklist of items for reports of case–control studies

Manuscript: Thiol–Disulfide Homeostasis and Erythrocyte Glutathione Balance in ST-Elevation Myocardial Infarction: A Prospective Case–Control Study

Journal: Journal of Cardiovascular Development and Disease (MDPI) — Article

Date: 1 August 2026

Reporting follows the STROBE recommendations. Locations refer to the numbered sections of the submitted manuscript file, which carries the author details, the ethics committee name, and the approval number and date in its back matter.

Item	Section/Topic	Recommendation	Location in manuscript
1	Title and abstract	(a) Study design in title/abstract; (b) informative, balanced abstract	Title; Abstract
2	Introduction — Background/rationale	Scientific background and rationale	Section 1 (Introduction), ¶1–3
3	Objectives	Specific objectives / hypotheses	Section 1 (Introduction), final ¶
4	Methods — Study design	Present key design elements early	Section 2.1 (Study Design and Setting)
5	Setting	Setting, locations, relevant dates	Section 2.1 (Study Design and Setting)
6	Participants	Eligibility, sources, case/control selection, rationale for case/control ratio	Section 2.2 (Participants)
7	Variables	Outcomes, exposures, predictors, confounders, effect modifiers	Section 2.3 (Variables and Measurements)
8	Data sources/measurement	Sources and details of assessment methods	Section 2.3 (Variables and Measurements) — validated automated assays
9	Bias	Efforts to address potential sources of bias	Section 2.2 (Participants, exclusions); Section 2.5 (adjusted and sensitivity analyses)
10	Study size	How study size was arrived at	Section 2.4 (Sample Size)
11	Quantitative variables	Handling of quantitative variables/groupings	Section 2.5 (Statistical Analysis)
12	Statistical methods	(a) Methods incl. confounding; (b) subgroups/interactions; (c) missing data; (d) matching; (e) sensitivity analyses	Section 2.5 (Statistical Analysis)
13	Results — Participants	Numbers at each stage; reasons for non-participation; flow diagram	Section 3.1 (Participants); Figure 1
14	Descriptive data	Characteristics; missing data per variable	Section 3.1 (Participants); Table 1
15	Outcome data	Numbers in each exposure category	Tables 1 and 2
16	Main results	(a) Estimates with 95% CI; (b) category boundaries; (c) absolute risk if relevant	Section 3.2; Table 2 (effect sizes with 95% CI)
17	Other analyses	Subgroups, interactions, sensitivity analyses	Sections 3.3–3.5; Figures 3 and 4
18	Discussion — Key results	Summarize key results with reference to	Section 4 (Discussion), ¶1

		objectives	
19	Limitations	Limitations, sources of bias, direction/magnitude	Section 4.1 (Strengths and Limitations)
20	Interpretation	Cautious overall interpretation	Section 4 (Discussion); Section 5 (Conclusions)
21	Generalisability	External validity	Section 4.1 (Strengths and Limitations) — single-center
22	Other information — Funding	Funding role	Funding statement (back matter)
