# Supplementary material for: Thiol–Disulfide Homeostasis and Erythrocyte Glutathione Balance in ST-Elevation Myocardial Infarction: A Prospective Case–Control Study
Source: J Cardiovasc Dev Dis. 2026 Sep 16;13(9):466. doi: 10.3390/jcdd13090466 (PMC13607282; doi:10.3390/jcdd13090466)
Supplement: Supplementary file 1 [file jcdd-13-00466-s001.zip › Supplementary Tables_S1_S5.pdf]

Supplementary Materials

Thiol–Disulfide Homeostasis and Erythrocyte Glutathione Balance in ST-Elevation Myocardial Infarction: A Prospective Case–Control Study

Table S1. Between-group differences in all ten redox markers after adjustment for age and sex, and after additional adjustment for smoking (linear models, n = 169).

Marker	Adjusted β for STEMI (95% CI), age and sex	p	Adjusted β for STEMI (95% CI), age, sex and smoking	p
Native thiol, μmol/L	−85.0 (−109.6 to −60.5)	< 0.001	−84.9 (−109.4 to −60.4)	< 0.001
Total thiol, μmol/L	−72.4 (−98.3 to −46.6)	< 0.001	−72.3 (−98.2 to −46.5)	< 0.001
Disulfide, μmol/L	6.29 (3.85 to 8.73)	< 0.001	6.28 (3.83 to 8.73)	< 0.001
Disulfide/native thiol ratio, %	3.31 (2.50 to 4.12)	< 0.001	3.30 (2.49 to 4.11)	< 0.001
Disulfide/total thiol ratio, %	2.60 (1.97 to 3.23)	< 0.001	2.59 (1.96 to 3.23)	< 0.001
Native thiol/total thiol ratio, %	−5.20 (−6.46 to −3.93)	< 0.001	−5.19 (−6.45 to −3.92)	< 0.001
Reduced glutathione, μmol/L	−127.6 (−159.4 to −95.8)	< 0.001	−127.7 (−159.6 to −95.8)	< 0.001
Total glutathione, μmol/L	−70.2 (−101.0 to −39.5)	< 0.001	−70.4 (−101.1 to −39.7)	< 0.001
Oxidized glutathione, μmol/L	28.7 (20.5 to 36.9)	< 0.001	28.6 (20.5 to 36.8)	< 0.001
Oxidized/reduced glutathione ratio	0.047 (0.035 to 0.060)	< 0.001	0.047 (0.035 to 0.060)	< 0.001

β is the adjusted mean difference for patients with ST-elevation myocardial infarction relative to controls. Models adjusted for comorbidity are not estimable, because hypertension, diabetes mellitus, hyperlipidemia and known cardiac disease were absent in every control by eligibility.

Table S2. Sex-stratified comparison of all ten redox markers between patients with ST-elevation myocardial infarction and controls.

Marker	Sex	Controls, n	STEMI, n	Control, median	STEMI, median	Rank-biserial r	p	Adjusted p
Native thiol, μmol/L	Male	15	68	380.7	312.6	−0.61	< 0.001	< 0.001
	Female	64	22	416.4	294.4	−0.64	< 0.001	< 0.001
Total thiol, μmol/L	Male	15	68	411.7	362.8	−0.46	0.006	0.006
	Female	64	22	454.0	348.9	−0.57	< 0.001	< 0.001
Disulfide, μmol/L	Male	15	68	14.9	21.9	0.61	< 0.001	< 0.001
	Female	64	22	17.5	23.8	0.46	0.001	0.002
Disulfide/native thiol ratio, %	Male	15	68	4.07	6.88	0.81	< 0.001	< 0.001
	Female	64	22	4.34	7.67	0.64	< 0.001	< 0.001
Disulfide/total thiol ratio, %	Male	15	68	3.76	6.05	0.81	< 0.001	< 0.001
	Female	64	22	3.99	6.66	0.64	< 0.001	< 0.001
Native thiol/total thiol ratio, %	Male	15	68	92.5	87.9	−0.81	< 0.001	< 0.001
	Female	64	22	92.0	86.7	−0.64	< 0.001	< 0.001

Reduced glutathione, μmol/L	Male	15	68	842.3	724.0	−0.65	< 0.001	< 0.001
	Female	64	22	889.1	726.6	−0.72	< 0.001	< 0.001
Total glutathione, μmol/L	Male	15	68	897.6	844.1	−0.40	0.017	0.017
	Female	64	22	936.9	860.8	−0.48	< 0.001	0.001
Oxidized glutathione, μmol/L	Male	15	68	25.6	53.5	0.55	< 0.001	0.001
	Female	64	22	27.4	61.8	0.75	< 0.001	< 0.001
Oxidized/reduced glutathione ratio	Male	15	68	0.031	0.069	0.67	< 0.001	< 0.001
	Female	64	22	0.030	0.085	0.80	< 0.001	< 0.001

Mann–Whitney U test within each sex stratum; adjusted p values are Benjamini–Hochberg false-discovery-rate adjusted across the 20 stratum-specific comparisons.

Table S3. Redox markers by sex within each group.

**Marker**	**Group**	**Men, n**	**Women, n**	**Men, median**	**Women, median**	**Rank-biserial r**	**p**	**Adjusted p**
Native thiol, μmol/L	STEMI	68	22	312.6	294.4	0.05	0.739	0.860
	Control	15	64	380.7	416.4	−0.31	0.064	0.643
Total thiol, μmol/L	STEMI	68	22	362.8	348.9	0.01	0.933	0.933
	Control	15	64	411.7	454.0	−0.36	0.033	0.643
Disulfide, μmol/L	STEMI	68	22	21.9	23.8	−0.14	0.313	0.712
	Control	15	64	14.9	17.5	−0.23	0.173	0.712
Disulfide/native thiol ratio, %	STEMI	68	22	6.88	7.67	−0.05	0.718	0.860
	Control	15	64	4.07	4.34	−0.14	0.392	0.712
Disulfide/total thiol ratio, %	STEMI	68	22	6.05	6.66	−0.05	0.721	0.860
	Control	15	64	3.76	3.99	−0.14	0.388	0.712
Native thiol/total thiol ratio, %	STEMI	68	22	87.9	86.7	0.05	0.718	0.860
	Control	15	64	92.5	92.0	0.14	0.392	0.712
Reduced glutathione, μmol/L	STEMI	68	22	724.0	726.6	−0.01	0.921	0.933
	Control	15	64	842.3	889.1	−0.23	0.171	0.712
Total glutathione, μmol/L	STEMI	68	22	844.1	860.8	−0.09	0.548	0.860
	Control	15	64	897.6	936.9	−0.20	0.243	0.712
Oxidized glutathione, μmol/L	STEMI	68	22	53.5	61.8	−0.19	0.195	0.712
	Control	15	64	25.6	27.4	0.05	0.774	0.860
Oxidized/reduced glutathione ratio	STEMI	68	22	0.069	0.085	−0.16	0.258	0.712
	Control	15	64	0.031	0.030	0.07	0.685	0.860

No marker differed by sex after adjustment in either group, the smallest adjusted p value being 0.643.

Table S4. Exploratory discrimination of single-axis and two-axis marker combinations, and correlation between the two axes.

Model	AUC (95% CI)	Compared with	DeLong p
GSH alone	0.863 (0.810 to 0.916)		
Native thiol alone	0.835 (0.775 to 0.896)		
GSH + native thiol	0.916 (0.876 to 0.956)	GSH alone	0.004
GSSG/GSH ratio + disulfide/total thiol	0.930 (0.894 to 0.965)	GSH alone	0.014

Logistic models fitted in the whole sample without prespecification, resampling or external validation; no diagnostic threshold is proposed.

Correlation between the serum and erythrocyte axes (Spearman, whole sample, n = 169).

Serum marker	Erythrocyte marker	rho	p
Native thiol, μmol/L	Reduced glutathione, μmol/L	0.446	< 0.001
Native thiol, μmol/L	Oxidized glutathione, μmol/L	−0.404	< 0.001
Native thiol, μmol/L	Oxidized/reduced glutathione ratio	−0.447	< 0.001
Disulfide, μmol/L	Reduced glutathione, μmol/L	−0.263	< 0.001
Disulfide, μmol/L	Oxidized glutathione, μmol/L	0.154	0.046
Disulfide, μmol/L	Oxidized/reduced glutathione ratio	0.192	0.013
Disulfide/total thiol ratio, %	Reduced glutathione, μmol/L	−0.421	< 0.001
Disulfide/total thiol ratio, %	Oxidized glutathione, μmol/L	0.323	< 0.001
Disulfide/total thiol ratio, %	Oxidized/reduced glutathione ratio	0.374	< 0.001

Moderate correlations indicate that the two axes carry related but not interchangeable information.

Table S5a. Correlations between the redox markers and peak troponin I on the raw and the log-transformed scale, within the ST-elevation myocardial infarction group (n = 90).

Marker	Spearman ϱ	Pearson r, raw scale	Pearson r, log scale (95% CI)	p, log scale	Adjusted p
Native thiol, μmol/L	0.05	0.05	0.09 (−0.11 to 0.30)	0.375	0.663
Total thiol, μmol/L	0.06	0.06	0.10 (−0.11 to 0.30)	0.372	0.663
Disulfide, μmol/L	−0.01	0.09	0.03 (−0.18 to 0.23)	0.809	0.809
Disulfide/native thiol ratio, %	−0.04	0.03	−0.06 (−0.26 to 0.15)	0.596	0.663
Disulfide/total thiol ratio, %	−0.04	0.03	−0.06 (−0.26 to 0.15)	0.570	0.663
Native thiol/total thiol ratio, %	0.04	−0.03	0.06 (−0.15 to 0.26)	0.568	0.663
Reduced glutathione, μmol/L	0.15	0.16	0.13 (−0.07 to 0.33)	0.207	0.663
Total glutathione, μmol/L	0.09	0.11	0.08 (−0.13 to 0.28)	0.458	0.663
Oxidized glutathione, μmol/L	−0.03	−0.10	−0.10 (−0.30 to 0.11)	0.338	0.663
Oxidized/reduced glutathione ratio	−0.07	−0.13	−0.12 (−0.32 to 0.09)	0.273	0.663

A monotone transformation leaves rank correlations unchanged, so the Spearman coefficients are identical on both scales and are reported once. Adjusted p values are Benjamini–Hochberg adjusted across the ten markers. No association reached significance on either scale.

Table S5b. Pairwise comparison of the single-marker areas under the curve against the marker with the highest value (DeLong test).

Comparison	AUC, reduced glutathione	AUC, comparator	Difference	p	Adjusted p
Native thiol, μmol/L	0.863	0.835	0.028	0.475	0.757
Total thiol, μmol/L	0.863	0.798	0.066	0.115	0.305
Disulfide, μmol/L	0.863	0.719	0.145	0.002	0.013
Disulfide/native thiol ratio, %	0.863	0.847	0.017	0.670	0.797
Disulfide/total thiol ratio, %	0.863	0.846	0.017	0.665	0.797
Native thiol/total thiol ratio, %	0.863	0.847	0.017	0.671	0.797
Total glutathione, μmol/L	0.863	0.757	0.107	< 0.001	< 0.001
Oxidized glutathione, μmol/L	0.863	0.822	0.042	0.251	0.497
Oxidized/reduced glutathione ratio	0.863	0.862	0.001	0.972	0.994

DeLong test for correlated curves, since every marker was measured in every participant. All 45 pairwise comparisons among the ten single markers were tested, and 8 reached significance after Benjamini–Hochberg adjustment across those comparisons. None of the significant comparisons lay among the five markers with the highest areas under the curve, which were statistically indistinguishable from one another, so the numerical ranking of the leading markers does not establish that any of them discriminates better than the others.
